# Ndfip1 Prevents Rotenone-Induced Neurotoxicity and Upregulation of α-Synuclein in SH-SY5Y Cells

**DOI:** 10.3389/fnmol.2020.613404

**Published:** 2021-01-05

**Authors:** Xin Liu, Le Qu, Na Zhang, Xiaoqi Yu, Zhixin Xiao, Limei Song, Junxia Xie, Huamin Xu

**Affiliations:** ^1^Shandong Provincial Key Laboratory of Pathogenesis and Prevention of Neurological Disorders and State Key Disciplines: Physiology, Department of Physiology, Medical College of Qingdao University, Qingdao, China; ^2^Institute of Brain Science and Disease, Qingdao University, Qingdao, China

**Keywords:** Parkinson's disease, α-synuclein, Ndfip1, rotenone, dopamine neuron

## Abstract

Nedd4 family interacting protein 1 (Ndfip1) is an adaptor of Nedd4-family ubiquitin ligases. Experimental results showed that Ndfip1 had a potential neuroprotective effect in neurology diseases. However, the neuroprotective effect and the underlying mechanisms of Ndfip1 in Parkinson's disease (PD) have not yet been fully elucidated. Therefore, in this study, we explored the neuroprotective effect of Ndfip1 against mitochondrial complex I inhibitor rotenone in a human dopaminergic neuroblastoma SH-SY5Y cell line and further elucidated its possible underlying mechanisms. Our results showed that rotenone could induce the up-regulation of α-synuclein (α-syn) in both mRNA and protein levels. The expression of Ndfip1 decreased at 24 h after rotenone treatment. Further study showed that high expression of Ndfip1 could protect SH-SY5Y cells against rotenone-induced neurotoxicity and antagonize the rotenone-induced increase in α-syn protein levels. In addition, high expression of Ndfip1 inhibited rotenone-induced increase in the protein levels of caspase-3 and decrease in tyrosine hydroxylase (TH). Further study showed that Ndfip1 did not affect the protein expression of iron regulatory protein 1 (IRP1), transferrin receptor 1 (TfR1), while antagonized the increase in protein levels of P62 and ferritin L caused by rotenone. Our findings provide specific identification of Ndfip1 proteins to inhibit the increase of α-syn in rotenone-induced SH-SY5Y cells. Ndfip1 might be a new theoretical drug target for the prevention and treatment of PD.

## Introduction

Parkinson's disease (PD) is a neurodegenerative disease caused by progressive degeneration of dopamine (DA) neurons in the substantia nigra (SN). Although the etiology and pathogenesis of PD have not yet been elucidated, many factors such as oxidative stress, inflammatory response, apoptosis, loss of mitochondrial function, and dysfunction of the ubiquitin-proteasome system are considered to be involved in the pathogenesis of PD. Alpha-synuclein (α-syn) is the major constituent of Lewy body, which is a pathological hallmark of PD and has been found in both familial and sporadic PD (Polymeropoulos et al., 1997; Kruger et al., 1998; Spillantini et al., 1998; Bucciantini et al., 2002; Lesage et al., 2013; Trinh and Farrer, 2013). Abnormal expression and aggregates of α-syn impaired a variety of cellular processes (Lashuel et al., 2002a,b; Kim et al., 2009; Colla et al., 2012; Choi et al., 2013), thus contributing to neurotoxicity (Chen et al., 2007; Tetzlaff et al., 2008; Nasstrom et al., 2011; Wang et al., 2011).

It has been shown that proteasome and lysosome play crucial roles in the degradation of misfolded and damaged proteins (Betarbet et al., 2005; Gan-Or et al., 2015). Failure of these pathways in the brain was associated with neuropathological disorders including PD (Moore et al., 2005). Nedd4 family interacting protein 1 (Ndfip1) is a transmembrane protein that contains 221 amino acid residues and has a molecular weight of approximately 26 kDa. It contains two PPxY motifs that bind to the WW domain of Nedd4 ubiquitin ligase to mediate ubiquitin-dependent protein degradation (Jolliffe et al., 2000). It has been reported that high levels of Ndfip1 as well as its binding protein Nedd4 were found in the surviving neurons after brain ischemia and traumatic brain injury and might be responsible for neuron survival (Sang et al., 2006; Howitt et al., 2012; Lackovic et al., 2012; Goh et al., 2014). Ndfip1 was also reported to protect neurons against Co^2+^ and Fe^2+^-induced neurotoxicity (Howitt et al., 2009). Our previous studies also found that Ndfip1 could mediate the degradation of divalent metal transporter 1 (DMT1) to antagonize Fe^2+^-induced neurotoxicity (Jia et al., 2015). All the above studies indicated that Ndfip1 might serve as an early sensor protein, which removes harmful misfolded proteins in damaged neurons and promotes the survival of neurons under stress conditions. Studies have shown that aggregation of α-syn in the SN of patients with PD was accompanied by an up-regulation of Ndfip1 (Howitt et al., 2014), but the relationship between them and the possible mechanisms of their actions in PD were not fully elucidated.

In this study, rotenone was used to induce PD cell model. Rotenone was a natural pesticide, which is a specific inhibitor of mitochondrial NADH dehydrogenase in respiratory chain complex I. It can cause oxidative stress and increased expression of α-syn (Huang et al., 2009; Chou et al., 2010; Deng et al., 2020). Therefore, real-time quantitative PCR, western blotting and immunofluorescence were performed on human neuroblastoma SH-SY5Y cells to study the neuroprotective effects and possible mechanisms of Ndfip1 on rotenone-induced neurotoxicity and increase in α-syn protein levels. The experimental results provide a new theoretical basis and therapeutic target for revealing the pathogenesis of PD.

## Materials and Methods

### Materials

The primary rabbit-anti-Ndfip1, rabbit-anti-tyrosine hydroxylase (TH) antibodies and rotenone were purchased from Sigma (St. Louis, MO, USA). The primary antibodies of rabbit-anti-α-syn, rabbit-anti-transferrin receptor 1 (TfR1), rabbit-anti-iron regulatory protein 1 (IRP1) and rabbit-anti-ferritin L were from Abcam (Cambridge, MA, USA). The primary antibodies of rabbit-anti-caspase-3 and rabbit-anti-P62 were from Cell Signaling Technology (Beverly, MA, USA). The monoclonal rabbit-anti-β-actin antibody was from Bioss (Beijing, China), and the goat anti-rabbit IgG labeled with HRP was from Santa Cruz (Dallas, TX, USA). The detailed information of antibodies are shown in Supplementary Table 1. High glucose/DMEM and FBS were from Hyclone (Logan, Utah, USA). Penicillin-streptomycin solution was bought from Beyotime (Shanghai, China). ECL ultrasensitive chemiluminescence kit was from MilliporeSigma (Billerica, MA, USA). All recombined adenoviruses were constructed by GeneChem (Shanghai, China). Other biological reagents and materials are from local commercial sources.

### Cell Culture

The SH-SY5Y cell line is purchased from the Shanghai Cell Bank of Chinese Academy of Sciences. The WT-α-syn iPC12 cells and A53T-α-syn iPC12 cells used in this experiment are a gift from Hong Kong Baptist University. SH-SY5Y cells, WT-α-syn iPC12 cells and A53T-α-syn iPC12 cells were cultured in high glucose/DMEM cell culture medium containing 10% fetal bovine serum (FBS) and 1% penicillin-streptomycin solution and maintained in a cell incubator with 5% CO_2_ and 95% air at 37°C. In the experiment, cells were cultured in plastic cell culture flasks at a density of 1 × 10^5^/cm^2^. Doxycline was added to WT-α-syn iPC12 cells and A53T-α-syn iPC12 cells to induce the expression of WT-α-syn and A53T-α-syn.

### Adenovirus Infection of SH-SY5Y Cells and Exposure to Rotenone

SH-SY5Y cells were seeded in 6-well plates at a density of 2 × 10^4^/cm^2^. When the cell density reached 50–70% confluence, the adenovirus with a multiplicity of infection (MOI) of 10 was then added to the medium to infect SH-SY5Y cells for 48 h. Fluorescence of green fluorescent protein (GFP) could be monitored to verify the infective efficiency. The recombinant adenovirus containing human Ndfip1 was named Ad.Ndfip1. Ad.GFP was used as an adenovirus control.

In this study, 300 nmol/L rotenone was used to detect the expression of target proteins (Sala et al., 2013; Li et al., 2014). SH-SY5Y cells were treated with 300 nmol/L rotenone for 3, 6, 9, 12, and 24 h separately. Then the RNA and protein were collected for the real-time quantitative PCR and western blots experiments to investigate the effect of rotenone on the expression of α-syn and Ndfip1. For the experiments of the neuroprotective effect of Ndfip1, cells were infected with Ad.GFP or Ad.Ndfip1 for 48 h following the treatment with 300 nmol/L rotenone for another 24 h.

### Real-Time PCR to Detect the mRNA Levels of Ndfip1 and α-syn

Total RNA was isolated by TRIZoL Reagent (Invitrogen, USA). Then 5 μg of total RNA was reversely transcribed by 20 μL reaction system using AMV reverse transcription system (Promega Corporation, USA). Real-time PCR was performed in a real-time PCR system (Eppendorf) using the SYBR Green (QIAGEN) dye. The following is the sequence of different primers: human Ndfip1 sense primer: 5′-CATCGAATCATTAGTGGTTA-3′; antisense primer: 5′-GATGGAGGATGAATAAAGC-3′. Human alpha-synuclein sense primer: 5′-CAGTGTATTTCAAAGTCTTC-3′; antisense primer: 5′-AGGTGTTTTAAGTTTCTTCTA-3′. Human β-actin sense primer: 5′- CATGTACGTTGCTATCCAGGC-3′; antisense primer: 5′- CTCCTTAATGTCACGCACGAT-3′. In this study, β-actin mRNA was used as a standard control. Amplification and detection were performed under the following conditions: 95° C for 30 s followed by 40 cycles of 95° C for 10 s and 60° C for 30 s. The 2^−ΔΔCt^ method was used in the calculation of relative mRNA expression levels.

### Protein Extraction and Western Blotting

After cells were treated with rotenone or/and infected by recombinant adenovirus, the cells were washed once with cold PBS, and then lysed on ice for 30 min in 100 μL of lysis solution by mixing strong RIPA lysis buffer with PMSF (Sigma, USA) in a 99:1 ratio. The lysate was then centrifuged and the protein-containing supernatant was collected. Next, the concentration of the protein was measured using the BCA protein concentration determination kit (Beyotime). Proteins with 5 × loading buffer (Beyotime) were incubated at 95°C for 10 min.

The total 20 μg protein was separated by 12–15% sodium dodecyl sulfate polyacrylamide gel electrophoresis and the protein in the gel was transferred to a PVDF membrane. After blocking, blots were probed with antibody of α-synuclein, caspase-3, TH, P62, TfR1, IRP1 and ferritin L (1: 1,000) or Ndfip1 antibody (1: 500). Rabbit monoclonal β-actin antibody (1: 10,000) was used as loading control. The goat anti-rabbit IgG labeled with HRP was used at 1: 10,000. ECL ultrasensitive chemiluminescence kit and UVP gel imaging system were used to observe the expression of proteins. During photographing, camera settings were used to optimize the exposure time and determine the appropriate final exposure conditions.

### Immunofluorescent and 4,6-Diamidino-2-phenylindole (DAPI) Staining

Cells were grown on glass coverslips and fixed in 4% paraformaldehyde for 30 min. Cells were then washed with 0.01 M PBS 3 times for 10 min and permeabilized with 0.1% Triton X-100 in 5% donkey serum-PBS for 1 h at room temperature. Subsequently, cells were incubated with rabbit anti-α-synuclein antibody (1:100) in PBS at 4°C overnight. After three PBS washes for 10 min, Alexa Flour568 conjugated goat anti-rabbit IgG secondary antibody (1:500) in PBS was added and incubated for 2 h at room temperature. Wash with PBS 3 times for 10 min. Nuclei were stained with DAPI at room temperature for 10 min in the dark and washed twice with PBS.

DAPI staining was also performed to assess apoptosis. Percentage of apoptotic cells with DNA fragmentation, nuclear condensation, and segmentation was counted manually by researchers blinded to the treatment schedule using a fluorescence microscope. The data were expressed as a percentage of apoptotic cells to the total number of cells.

### Statistical Analysis

The experimental results were analyzed by GraphPad Prism 5 statistical software and data was expressed as mean ± S.E.M. One-way analysis of variance (ANOVA) was used to compare data. *P* < 0.05 was considered to be statistically significant.

## Results

### The Expressions of α-syn and Ndfip1 After Exposure to Rotenone in SH-SY5Y Cells

**Figure 1:**
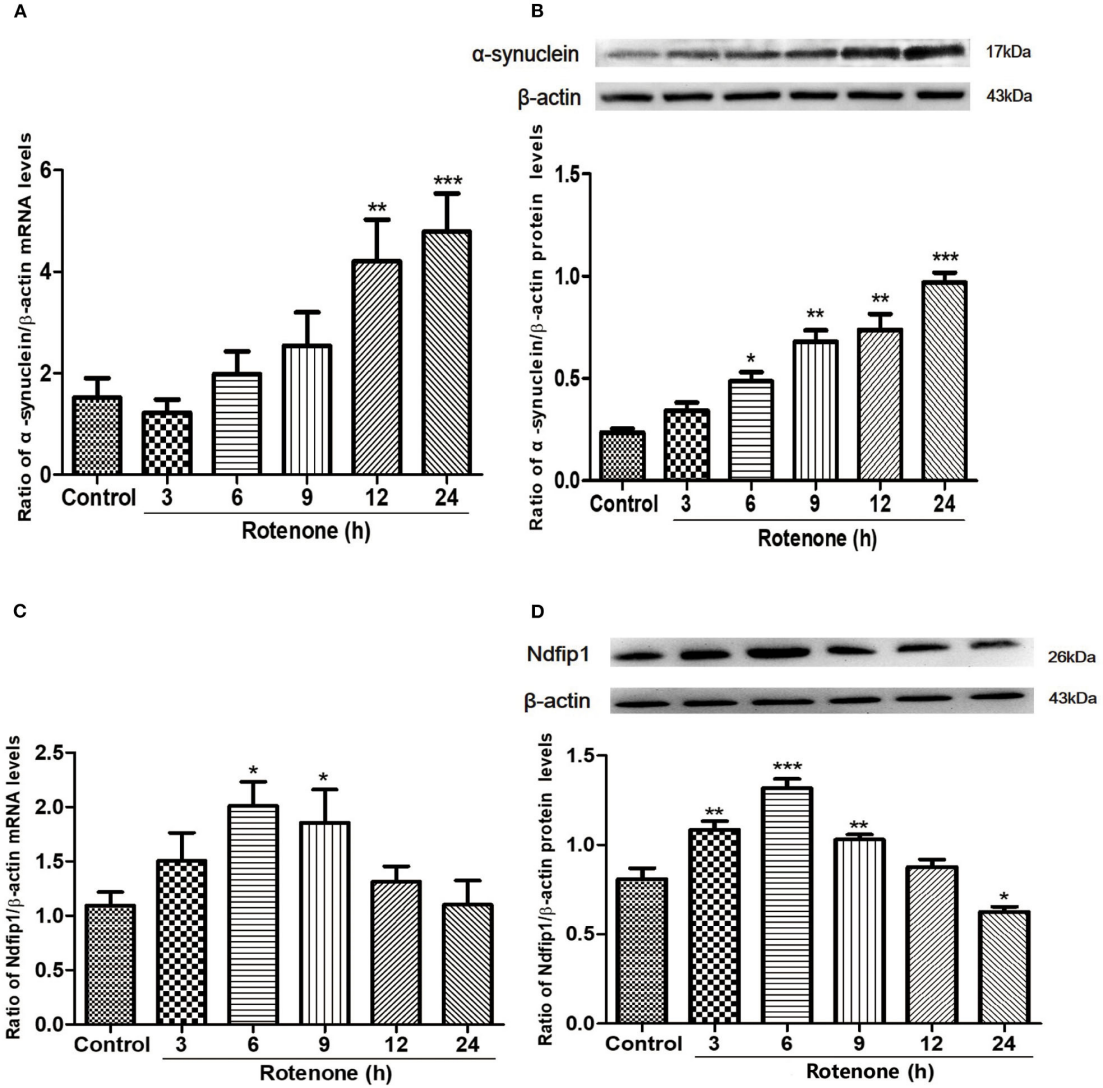
The mRNA and protein expressions of α-syn and Ndfip1 after exposure to rotenone in SH-SY5Y cells. **(A)** The mRNA expression of α-syn in rotenone-treated SH-SY5Y cells increased at 12 and 24 h compared with the control (**P < 0.01, ***P < 0.001, compared with the control). **(B)** The protein expression of α-syn increased at 6, 9, 12, and 24 h after rotenone exposure compared with the control in SH-SY5Y cells (*P < 0.05, **P < 0.01, ***P < 0.001, compared with the control). **(C)** The mRNA expression of Ndfip1 in rotenone-treated SH-SY5Y increased at 6 and 9 h, compared with the control (*P < 0.05, compared with the control). **(D)** The protein expression of Ndfip1 increased significantly at 3, 6, and 9 h and decreased at 24 h after rotenone exposure in SH-SY5Y cells, compared with the control (*P < 0.05, **P < 0.01, ***P < 0.001, compared with the control).

Following treatment with 300 nmol/L rotenone, the mRNA and protein levels of α-syn in SH-SY5Y cells were quantified by real-time PCR and western blots in different time point. Results showed that 300 nmol/L rotenone could significantly increase the mRNA expression of α-syn at 12 and 24 h compared with the control (Figure 1A). We then detected the protein levels of α-syn in different time point after exposure to rotenone. Results showed that the protein expression of α-syn increased from 6 h after rotenone treatment and increased further at 9, 12, and 24 h after rotenone exposure in SH-SY5Y cells (Figure 1B).

To investigate whether rotenone treatment could affect the expression of Ndfip1, we detected the expression of Ndfip1 in mRNA and protein levels. Data showed that the mRNA expression of Ndfip1 increased at 6 and 9 h after exposure to rotenone, compared with the control (Figure 1C). In addition, we also detected the protein levels of Ndfip1 in rotenone-treated SH-SY5Y cells by western blots. Data showed that the protein expression of Ndfip1 increased significantly at 3, 6, 9 h and decreased at 24 h after rotenone treatment in SH-SY5Y cells, compared with the control (Figure 1D).

### High Levels of Ndfip1 Protected SH-SY5Y Cells Against Rotenone-Induced Morphological Change

**Figure 2:**
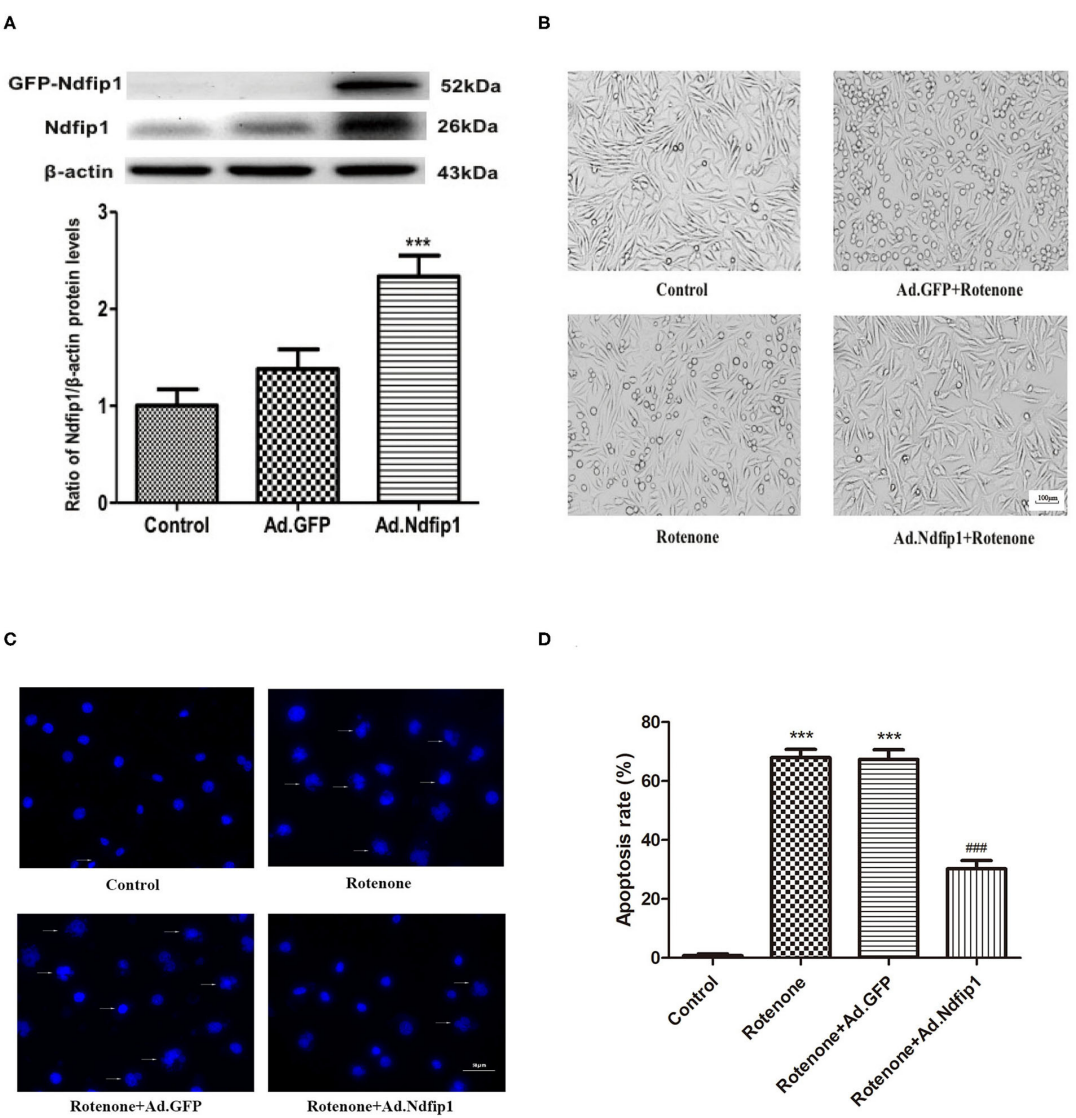
The effect of high levels of Ndfip1 on rotenone-induced morphological change and apoptosis. **(A)** The expression of Ndfip1 increased significantly in the SH-SY5Y cells with infection of Ad.Ndfip1 compared with Ad.GFP infected cells and the normal controls (***P < 0.001, compared with the control). **(B)** Cells were attached with the regular shape in the control group. After exposure to 300 nmol/L rotenone, cell bodies showed shrinkage and detachment. High levels of Ndfip1 significantly inhibited the rotenone-induced morphological damage. In addition, there were not significant morphology changes in Ad.GFP infected cells compared with the control. Scale bar indicates 100 μm. **(C)** Representative fluorescence photographs show the nuclei morphology in different groups. Arrows indicate perinuclear apoptotic bodies and nuclear fragmentation typically observed in apoptotic cells. **(D)** The percentage of apoptotic cells was calculated as the ratio of apoptotic cells to total cells. Results are expressed as mean ± S.E.M. of three independent experiments (***P < 0.001, compared with control; ^###^*P* < 0.001, compared with rotenone group and Ad.GFP/rotenone group). Scale bar indicates 50 μm.

In this study, recombinant adenovirus-induce overexpression of Ndfip1 was used. The expression of Ndfip1 was detected after Ad.Ndfip1 or Ad.GFP infection in SH-SY5Y cells for 48 h. We have detected two bands of Ndfip1 in 26 KD and 52 KD (GFP-fusion) in this experiment. Data showed that the expression of Ndfip1 increased significantly in the SH-SY5Y cells with infection of Ad.Ndfip1 compared with Ad.GFP infected cells and the normal controls (Figure 2A).

To investigate whether the high levels of Ndfip1 could protect SH-SY5Y cells against rotenone, we observed morphological change by phase-contrast imaging after different treatment. As shown in Figure 2B, cells were attached with the regular shape in the control group. After exposure to 300 nmol/L rotenone, cell bodies showed shrinkage and detachment. Furthermore, DAPI staining showed that nuclei of control cells were round and large in size. However, the nuclei of cells with rotenone treatment appeared hypercondensed and perinuclear apoptotic bodies; Overexpression of Ndfip1 significantly protected against rotenone-induced nuclear condensation and perinuclear apoptotic bodies in SH-SY5Y cells (Figure 2C). The percentage of apoptotic cells increased in the rotenone-treated cells, which could be inhibited by overexpression of Ndfip1 (Figure 2D).

### High Levels of Ndfip1 Protected SH-SY5Y Cells Against Rotenone-Induced Decrease in TH Expression and Increase in Caspase-3 Expression

**Figure 3:**
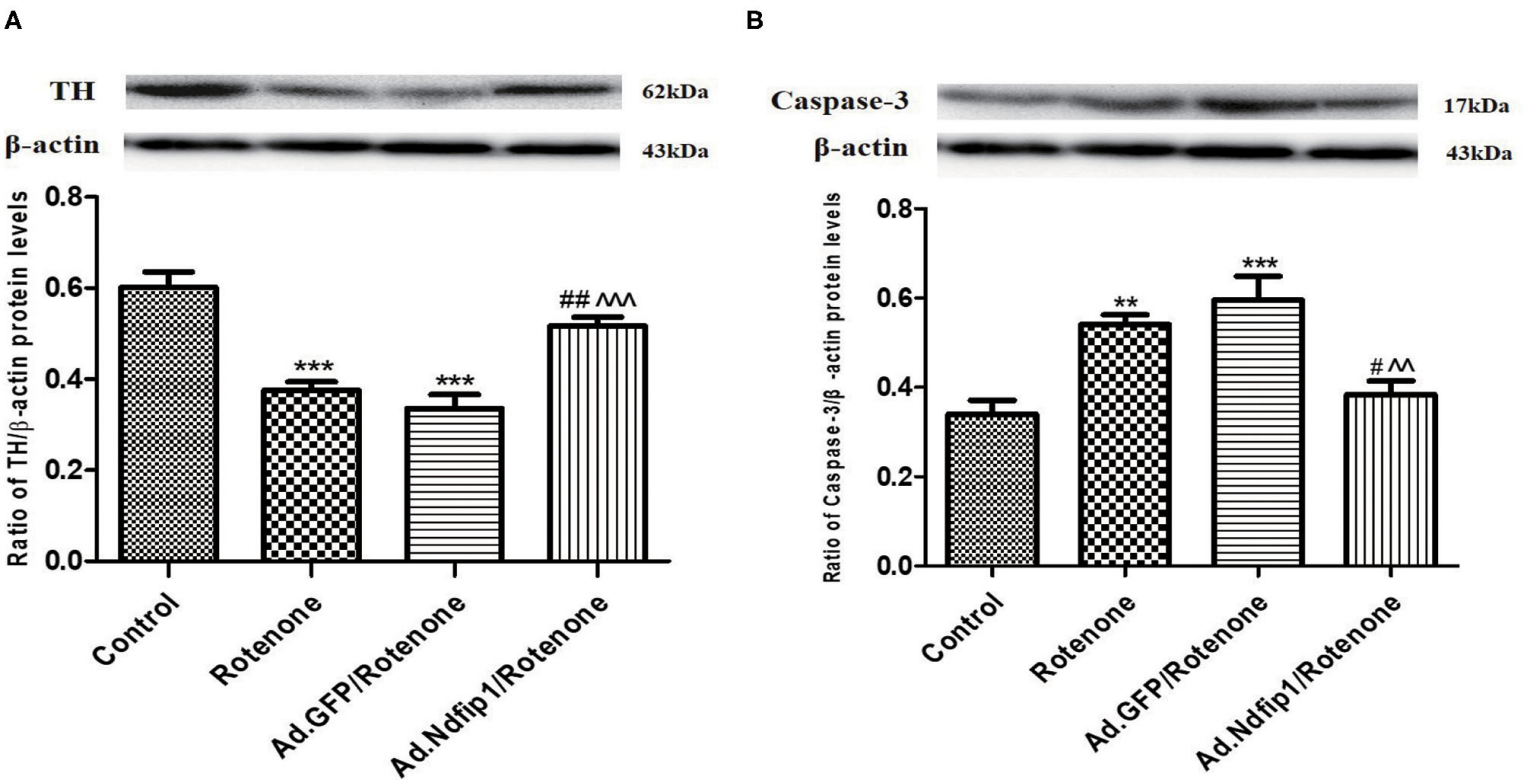
Ndfip1 antagonized rotenone-induced down-regulation of TH and increase of caspase-3 in SH-SY5Y cells. **(A)** Ndfip1 antagonized down-regulation of TH induced by rotenone in SH-SY5Y cells. Western blots were applied to detect the protein levels of TH in various groups. Compared with the control group, treatment of 300 nmol/L rotenone for 24 h resulted in down-regulation of TH protein in SH-SY5H cells. The protein levels of TH in Ad.Ndfip1/rotenone group were increased compared to the Ad.GFP/rotenone group and rotenone group (***P < 0.001, compared with control; ^##^*P* < 0.01, compared with rotenone group; ^∧∧∧^*P* < 0.001, compared with Ad.GFP/rotenone group; *n* = 6). **(B)** Ndfip1 antagonized rotenone-induced caspase-3 activation in SH-SY5Y cells. Western blots were applied to detect the protein levels of caspase-3 in various groups. Compared with the control group, treatment of 300 nmol/L rotenone for 24 h resulted in up-regulation of caspase-3 protein in SH-SY5H cells. The protein levels of caspase-3 in Ad.Ndfip1/rotenone group were reduced compared to the Ad.GFP/rotenone and rotenone group (**P < 0.01, ***P < 0.001, compared with control; ^#^*P* < 0.05, compared with rotenone group; ^∧∧^*P* < 0.01, compared with Ad.GFP/rotenone group; *n* = 6).

After SH-SY5Y cells were infected with recombinant adenovirus for 24 h and then incubated with 300 nmol/L rotenone for another 24 h, the protein expression of TH and caspase-3 were detected. Results showed that the protein levels of TH in rotenone and Ad.GFP/rotenone group were down-regulated by 37.5 and 44.2%, respectively, compared with the control group. Further study demonstrated that the expression of caspase-3 protein in rotenone group and Ad.GFP/rotenone group were up-regulated by 60.3 and 75.4%, respectively, compared with the control group. High levels of Ndfip1 could inhibit rotenone-induced down-regulation of TH (Figure 3A) and up-regulation of caspase-3 (Figure 3B).

### High Levels of Ndfip1 Dramatically Inhibited Rotenone-Induced Increase in the Protein Levels of α-syn in SH-SY5Y Cells

**Figure 4:**
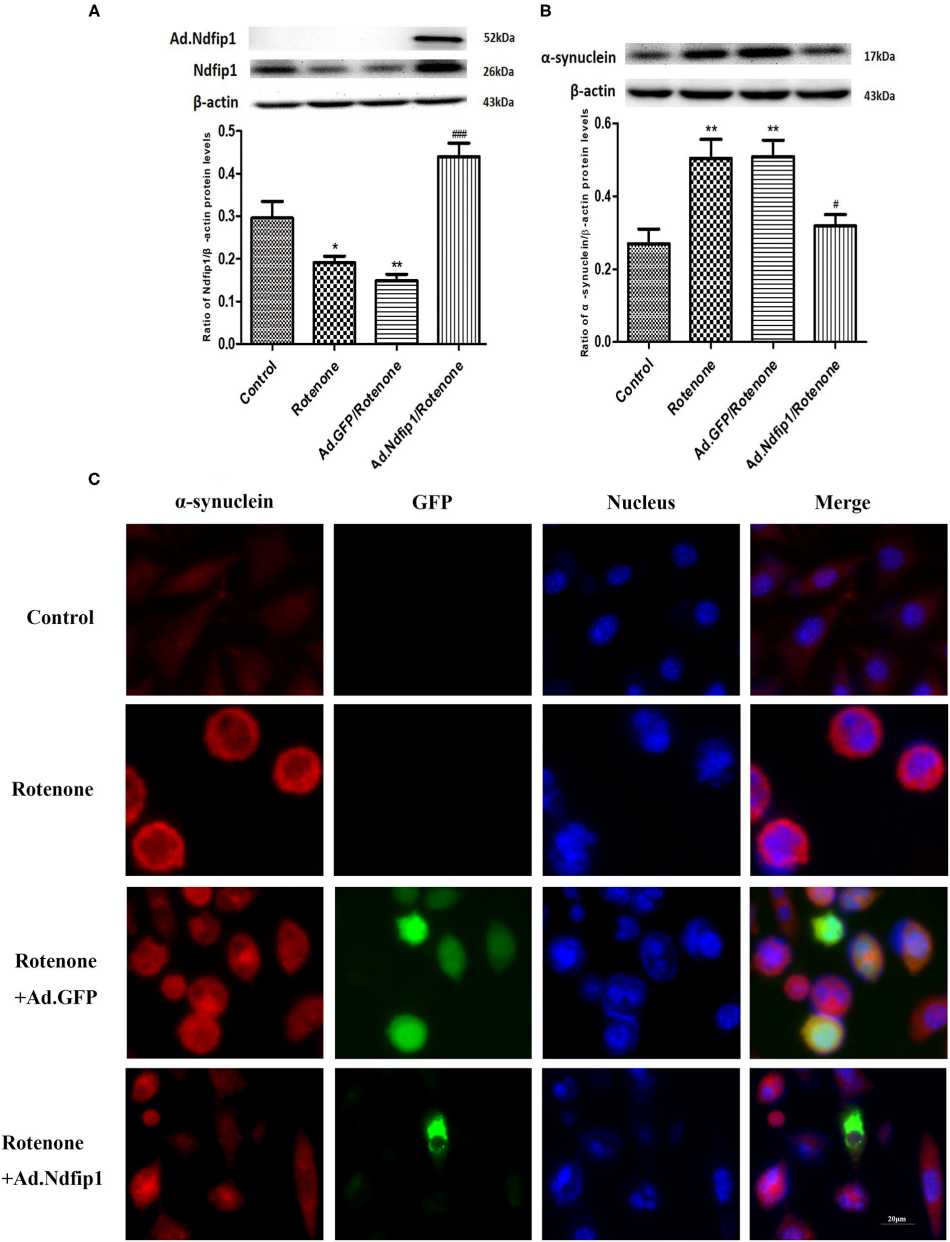
The effect of high levels of Ndfip1 on rotenone-induced increase of α-syn in SH-SY5Y cells. **(A)** The expression of Ndfip1 in rotenone-treated cells decreased significantly compared with the control. The higher protein levels of Ndfip1 were detected in cells infected with Ad.Ndfip1 following rotenone treatment, compared with rotenone or Ad.GFP/rotenone groups (*P < 0.05, **P < 0.01, compared with the control; ^###^*P* < 0.001, compared with rotenone-treated cells). **(B)** The expression of α-syn in rotenone-treated cells increased significantly compared with the control. Ad.Ndfip1 infection could inhibit rotenone-induced increase in the protein levels of α-syn. Additionally, Ad.GFP treatment had no apparent effect on rotenone-induced increase in the protein levels of α-syn (**P < 0.01, compared with the control; ^#^*P* < 0.05, compared with rotenone-treated cells). **(C)** Immunofluorescence with anti-α-syn antibodies indicated the basal levels of α-syn expression (red fluorescence) in cultured SH-SY5Y cells and increased expression in rotenone and Ad.GFP/rotenone cells, but down-regulation in Ad.Ndfip1/rotenone cells. Scale bar indicates 20 μm.

Rotenone exposure for 24 h decreased the expression of Ndfip1 in SH-SY5Y cells. Ad.Ndfip1 infection induced an obvious increase in the protein levels of Ndfip1 after exposure to rotenone (Figure 4A). To verify whether high levels of Ndfip1 could protect SH-SY5Y cells against rotenone-induced increase in α-syn, the α-syn protein levels were detected by western blots. Results showed that the protein levels of α-syn were obviously decreased in Ad.Ndfip1 infected cells compared with Ad.GFP infected cells and control cells after rotenone treatment, as shown in Figure 4B. Additionally, the study of immunofluorescence indicated the basal levels of α-syn expression (red fluorescence) in cultured SH-SY5Y cells and increased expression in rotenone-treated cells, but down-regulation in Ad.Ndfip1/rotenone cells (Figure 4C). These results demonstrate a role for Ndfip1 in regulating α-syn protein levels in rotenone-treated SH-SY5Y cells.

### High Levels of Ndfip1 Did Not Affect α-Syn Protein Expression in WT-α-syn iPC12 Cells and A53T-α-syn iPC12 Cells Treated With Doxycline (DOX)

**Figure 5:**
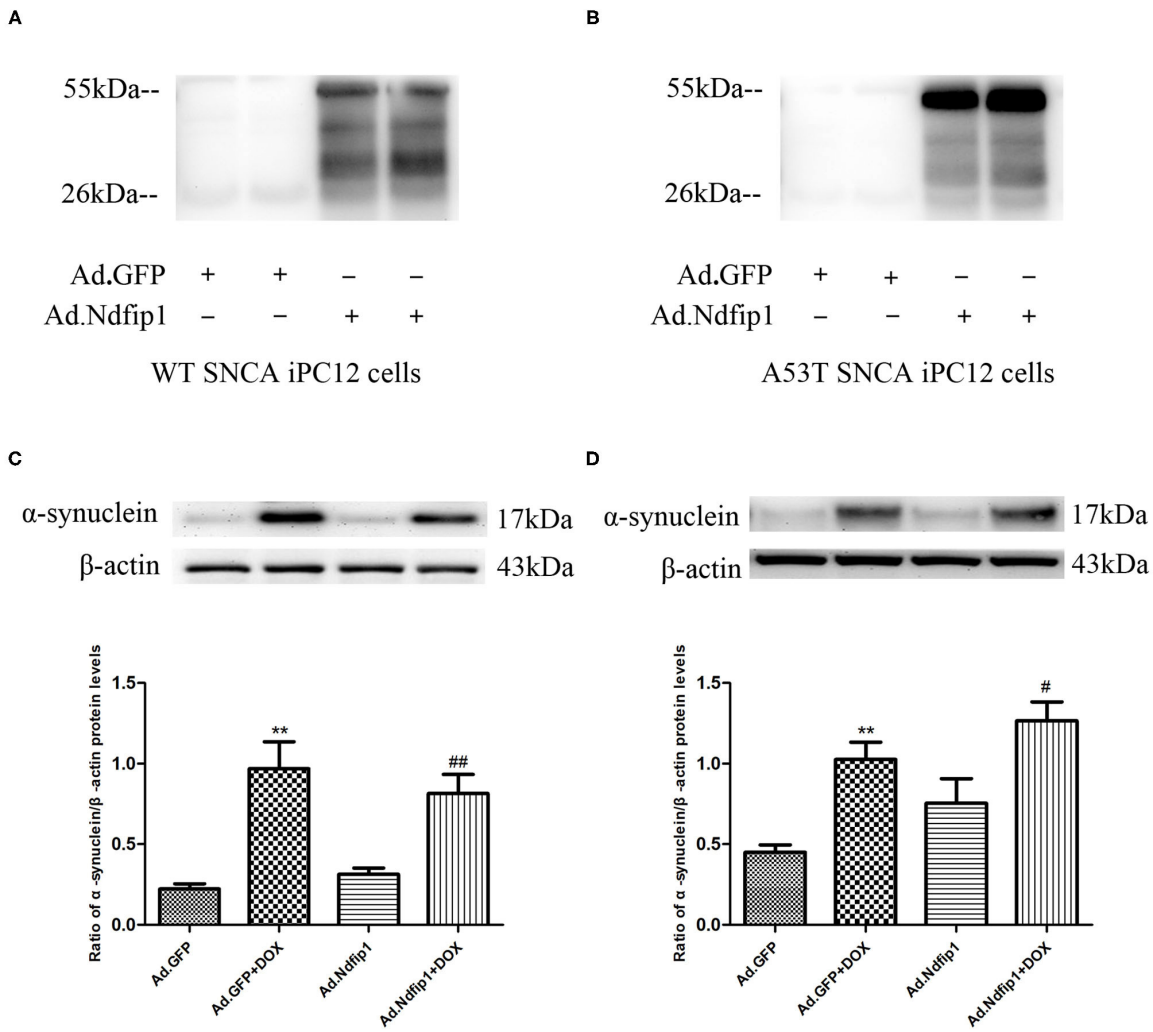
The effect of Ndfip1 on α-syn protein expression in WT-α-syn iPC12 cells and A53T-α-syn iPC12 cells. **(A)** Increased expression of Ndfip1 was observed in Ad.Ndfip1 group in WT-α-syn iPC12 cells. **(B)** Increased expression of Ndfip1 was observed in Ad.Ndfip1 group in A53T-α-syn iPC12 cells. **(C)** Increased expression of α-Syn was observed in Ad.Ndfip1 + DOX and Ad.GFP + DOX group. The expression of α-syn did not change in the Ad.Ndfip1 + DOX group, compared with the Ad.GFP+DOX group in WT-α-syn iPC12 cells. β-actin was used as a loading control (**P < 0.01, compared with the Ad.GFP group; ^##^*P* < 0.01, compared with Ad.Ndfip1 group). **(D)** Increased expression of α-Syn was observed in Ad.Ndfip1 + DOX and Ad.GFP + DOX group. The expression of α-syn did not change in the Ad.Ndfip1 + DOX group, compared with the Ad.GFP + DOX group in A53T-α-syn iPC12 cells. β-actin was used as a loading control (**P < 0.01, compared with the Ad.GFP group; ^#^*P* < 0.05, compared with Ad.Ndfip1 group).

The recombinant adenovirus carrying Ndfip1 was used to infect A53T-α-syn iPC12 cells or WT-α-syn iPC12 cells. The expression of Ndfip1 was detected 48 h after infection. The experimental results showed that the expression of Ndfip1 increased significantly in Ad.Ndfip1 infected iPC12 cells, compared with the Ad.GFP group (Figures 5A,B). Then after infection of A53T-α-syn iPC12 cells or WT-α-syn iPC12 cells with Ad.Ndfip1 or Ad.GFP for 24 h, doxycline was added to iPC12 cells for another 24 h to induced the expression of α-syn. Western blots were used to detect the changes of α-syn. The results showed that the expression of α-syn did not change in the Ad.Ndfip1 + DOX group in A53T-α-syn iPC12 cells or WT-α-syn iPC12 cells, compared with the Ad.GFP+DOX group (Figures 5C,D).

### High Levels of Ndfip1 Antagonized Rotenone-Induced Increase in P62 and Ferritin L Protein Expression

**Figure 6:**
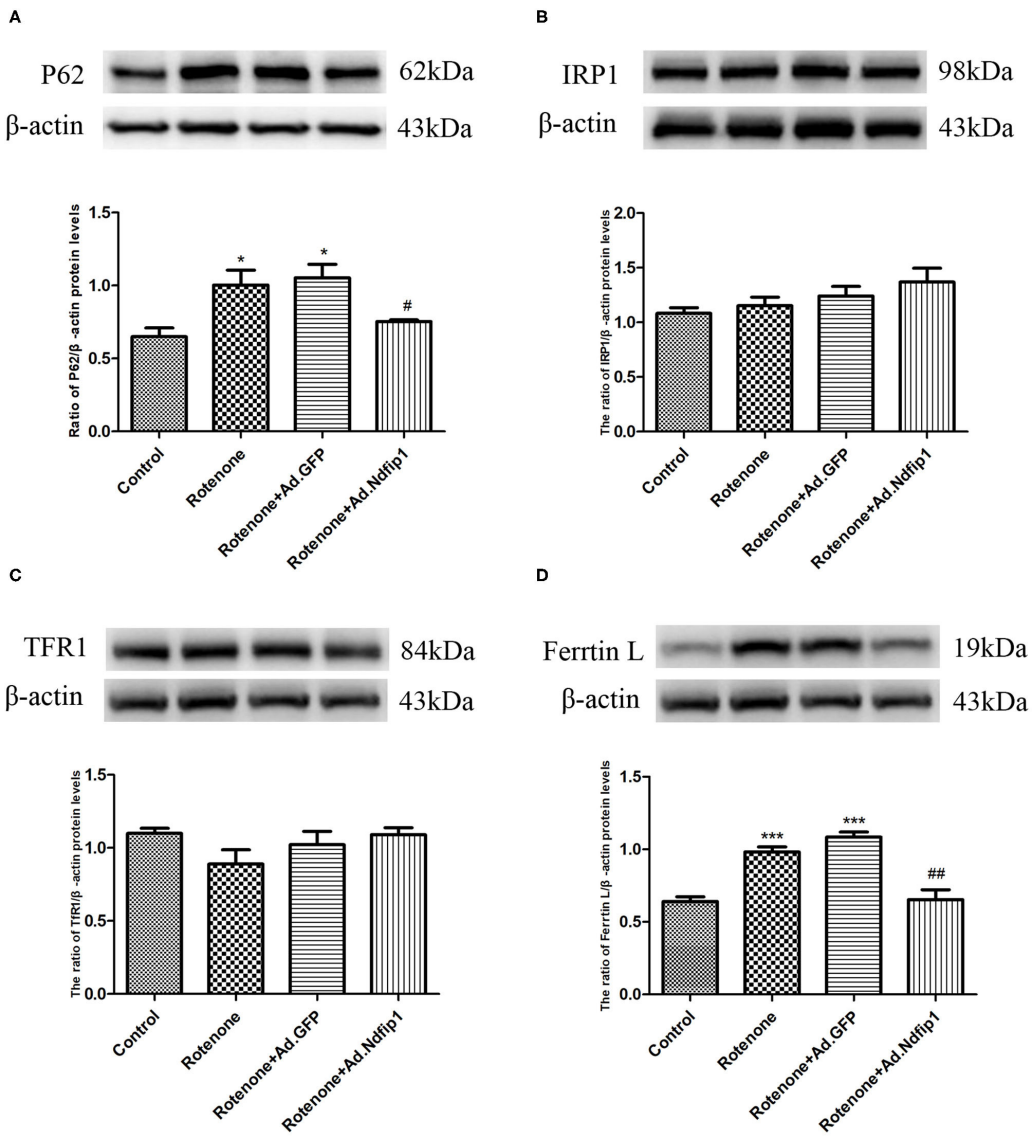
High levels of Ndfip1 antagonized rotenone-induced increase in P62 and ferritin L protein expression. **(A)** The protein expression of P62 in the rotenone group up-regulated significantly, compared with the control group. High level of Ndfip1 could antagonize rotenone-induced up-regulation of P62 (*P < 0.05, compared with the control; ^#^*P* < 0.05, compared with rotenone-treated cells). **(B)** Changes in the protein expression of IRP1. **(C)** Changes in the protein expression of TfR1. **(D)** The protein expression of ferritin L in the rotenone group up-regulated significantly, compared with the control group. High level of Ndfip1 could antagonize rotenone-induced up-regulation of ferritin L (***P < 0.001, compared with the control; ^##^*P* < 0.01, compared with rotenone-treated cells).

To further investigate the possible mechanisms underlying the protective effect of Ndfip1 against rotenone, we detect the expression of the autophagy-related protein P62. The results showed that the expression of P62 protein in the rotenone group up-regulated significantly, compared with the control group, which could be antagonized by high level of Ndfip1 (Figure 6A). In addition, we have tested the expression of iron-related proteins including iron regulatory protein 1 (IRP1), transferrin receptor 1 (TfR1), and ferritin L in cells treated with rotenone. The results showed that high expression of Ndfip1 did not affect the protein expression of IRP1, TfR1, while antagonized the increase in ferritin L protein expression caused by rotenone (Figures 6B–D).

## Discussion

In this study, we showed that the expression of Ndfip1 decreased and α-syn increased in rotenone-induced PD cell models. Further studies showed that high expression of Ndfip1 could protect SH-SY5Y cells against rotenone-induced neurotoxicity by inhibiting the up-regulation of caspase-3 and α-syn. However, high expression of Ndfip1 did not affect the α-syn protein level in DOX-induced A53T-α-syn iPC12 cells or WT-α-syn iPC12 cells. In addition, high expression of Ndfip1 inhibited rotenone-induced increase of P62 and ferritin L protein. This indicated that high expression of Ndfip1 might be a new theoretical target for the prevention and treatment of PD.

Alpha-syn aggregate is a critical event in the death of DA neurons and the progression of PD (Sanders and Greenamyre, 2013). Rotenone is an insecticide, which could inhibit complex I activity and promote α-syn up-regulation and aggregate (Betarbet et al., 2000; Sanders and Greenamyre, 2013; Chen et al., 2018). Our results also indicate that rotenone is able to increase both the mRNA and protein levels of α-syn in SH-SY5Y cells. Moreover, our results provide the first evidence for a role of rotenone in the regulation of Ndfip1. The analysis of Ndfip1 mRNA levels performed in this study demonstrates that rotenone leads to a transcriptional up-regulation of Ndfip1 after exposure to rotenone for 6 and 9 h. Data also shows that the protein expression of Ndfip1 increased significantly at 3, 6, and 9 h after rotenone treatment in SH-SY5Y cells. Other study has also shown that aggregation of α-syn in the SN of PD patients was accompanied by an up-regulation of Ndfip1 (Howitt et al., 2014). It was reported that Ndfip1 is strongly expressed in surviving neurons around the site of injury following transient focal cerebral ischemia or traumatic brain injury to provide neuroprotection (Sang et al., 2006; Lackovic et al., 2012). This suggested that the up-regulation of Ndfip1 in rotenone-induced PD cell model might represent an adaptive or protective response to provide neuroprotection against rotenone. However, protein expression of Ndfip1 decreased at 24 h after rotenone treatment in SH-SY5Y cells. This decrease of Ndfip1 protein might result in the loss of its neuroprotection on rotenone-treated SH-SY5Y cells.

Ndfip1 was reported previously to have a neuroprotective effect on neurons (Sang et al., 2006; Lackovic et al., 2012; Jia et al., 2015; Liu et al., 2015). The above results in this study indicated that the decreased Ndfip1 might be involved in loss of neuroprotection in rotenone-treated SH-SY5Y cells. Then increasing Ndfip1 levels might be protective to rotenone-treated SH-SY5Y cells. Therefore, Ad.Ndfip1-induced high expression of Ndfip1 was used to investigate its neuroprotective effect against rotenone in this study. We first observed morphological change after different treatment. Results showed that Ad.Ndfip1-induced high levels of Ndfip1 significantly inhibited the rotenone-induced morphological damage and apoptosis. TH is the rate-limiting enzyme in the biosynthesis of DA and used as a marker of dopaminergic neurons. In this study, high levels of Ndfip1 could inhibit rotenone-induced decrease in TH protein levels. In addition, we also showed that Ndfip1 antagonized rotenone-induced increase in apoptosis protein caspase-3, further indicating the protective effect of high levels of Ndfip1 against rotenone-induced neurotoxicity.

Furthermore, our results showed that rotenone increased the expression of α-syn in SH-SY5Y cells. Then we further investigated whether Ndfip1 could affect rotenone-induced increase in α-syn expression in SH-SY5Y cells. Results showed that rotenone treatment increased α-syn significantly, which could be inhibited by high levels of Ndfip1. In addition, we also observed the effect of Ndfip1 on α-syn protein levels in A53T-α-syn iPC12 cells or WT-α-syn iPC12 cells, which express high levels of WT α-syn or A53T α-syn. Results shows that high expression of Ndfip1 does not affect α-syn protein level in WT SNCA iPC12 cells and A53T SNCA iPC12 cells. This indicated that the mechanisms underlying the effect of Ndfip1 on α-syn expression might be diverse response to different treatment. It has been shown that rotenone induces autophagy inhibition. In the present study, we demonstrated that rotenone treatment resulted in an elevated P62 level in SH-SY5Y cells in agreement with the previous study, indicating the autophagy inhibition of rotenone. High levels of Ndfip1 antagonized rotenone-induced elevation of P62 level. This suggests that the activation of autophagy flux might account at least partially for the effect of Ndfip1 against rotenone-induced upregulation of α-syn by increasing the degradation of α-syn.

In this study, we have also shown that the protein expression of iron-related proteins including IRP1 and TfR1 was not changed, while ferritin protein expression increased after rotenone treatment. IRP1 was known to regulate the expression of iron-related proteins including TfR1 and ferritin at translational level via binding iron responsive element (IRE) on these iron-related proteins. Our result suggested that rotenone-induced increase in ferritin protein expression is not dependent on IRP1/IRE system. This is consistent with the result showing that transient translational repression of ferritin synthesis via IRP was not involved in the regulation of ferritin protein level during rotenone treatment (MacKenzie et al., 2008). They also showed that ferritin transcription was activated by rotenone via an oxidative stress-mediated pathway leading to activation of ARE. It has been confirmed the regulation of ferritin-L by a MARE/ARE DNA sequence (Hintze and Theil, 2005). Our results in this study demonstrate that rotenone increases the expression ferritin L, which could be inhibited by high level of Ndfip1. This suggested that the effect of Ndfip1 might be partially through antagonizing oxidative stress-mediated activation of ARE cause by rotenone, thus involved in the regulation of ferritin-L.

## Conclusion

In conclusion, our study provides the possibility that the neuroprotective property of Ndfip1 on dopaminergic cells might be associated with decreased the protein levels of α-syn resulting from rotenone treatment. We also show that Ndfip1 has a protective effect on rotenone-induced PD cell model though antagonizing autophagy inhibition, apoptosis and oxidative stress caused by rotenone. The experimental study provides further insights and new experimental basis into the neurotoxicity caused by the abnormal accumulation of α-syn in PD and provides new experimental evidence for the treatment of PD.

## Data Availability Statement

The raw data supporting the conclusions of this article will be made available by the authors, without undue reservation.

## Author Contributions

HX and JX designed the experiments. HX and XL wrote the initial draft of the manuscript. XL, LQ, NZ, XY, ZX, and LS performed the experiments and analyzed the data. JX revised the manuscript. All authors have read and approved the final version of the manuscript.

## Conflict of Interest

The authors declare that the research was conducted in the absence of any commercial or financial relationships that could be construed as a potential conflict of interest.
