# Supplementary figures and images for: Ndfip1 Prevents Rotenone-Induced Neurotoxicity and Upregulation of α-Synuclein in SH-SY5Y Cells
Source: Front Mol Neurosci. 2021 Jan 5;13:613404. doi: 10.3389/fnmol.2020.613404 (PMC7813998; doi:10.3389/fnmol.2020.613404)

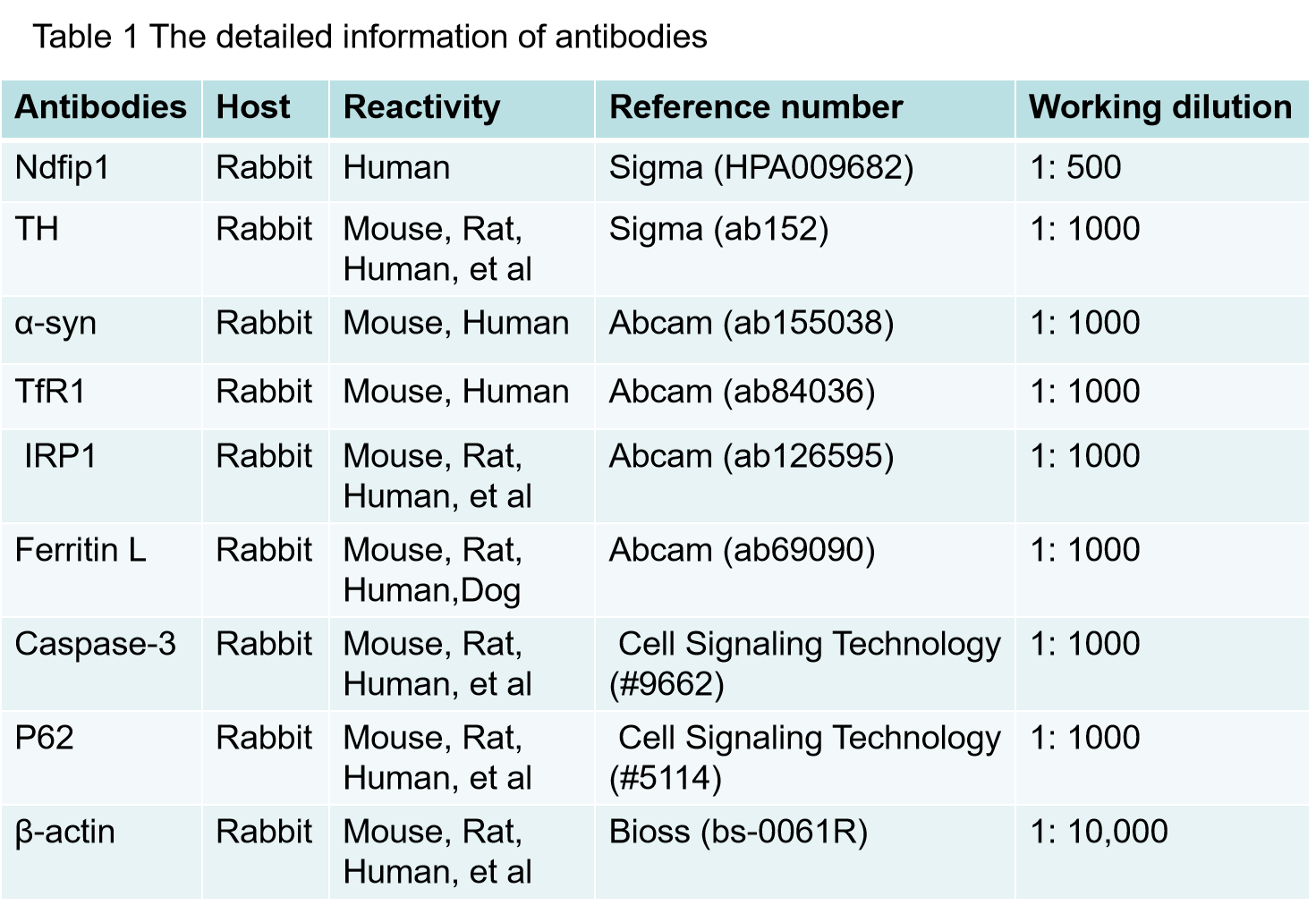

Supplement: Supplementary file 1 [file Image_1.TIF]
